# The 40bp indel polymorphism of *MDM2* increase the risk of cancer: An updated meta-analysis

**DOI:** 10.22099/mbrc.2019.31527.1364

**Published:** 2019-03

**Authors:** Abdolkarim Moazeni-Roodi, Saeid Ghavami, Mohammad Hashemi

**Affiliations:** 1 Department of Clinical Biochemistry, Iranshahr University of Medical Sciences, Iranshahr, Iran; 2 Department of Human Anatomy and Cell Science, Max Rady College of Medicine, Rady Faculty of Health Sciences, University of Manitoba, Winnipeg, MB, Canada; 3 Research Institute in Oncology and Hematology, CancerCare Manitoba, University of Manitoba, Canada; 4 Genetics of Non-communicable Disease Research Center, Zahedan University of Medical Sciences, Zahedan, Iran; 5 Department of Clinical Biochemistry, School of Medicine, Zahedan University of Medical Sciences, Zahedan, Iran

**Keywords:** MDM2, Indel, Polymorphism, rs3730485, Cancer

## Abstract

This meta-analysis aimed to provide an up-to-date comprehensive evaluation on the association between the *MDM2* 40bp indel polymorphism and cancer susceptibility. Eligible studies were retrieved by searching Web of Science, PubMed, Scopus, and Google scholar databases up to August 27, 2018. The pooled odds ratios (ORs) with 95% confidence intervals (CIs) were calculated to estimate the strength of association between the polymorphism and cancer risk. The findings of this meta-analysis revealed that the 40bp indel polymorphism significantly increased the risk of overall cancer risk in heterozygous (OR=1.06, 95%CI=1.01-1.11, P=0.016) and ID+DD (OR=1.07, 95%CI=1.01-1.14, P=0.027) genotypes. Stratified analysis by cancer type proposed that the study indel variant significantly associated with the risk of gastrointestinal cancer in heterozygous (OR=1.18, 95%CI=1.06-1.32, P=0.003) and ID+DD (OR=1.18, 95%CI=1.06-1.30, P=0.002) genotypes. The present findings showed a significant association between the *MDM2 *40bp indel polymorphism and overall cancer risk as well as gastrointestinal cancer susceptibility. Larger and well-designed researches are required to validate the findings association in detail.

## INTRODUCTION

Cancer remains one of the main leading cause of morbidity and mortality and poses a serious challenge to global public health worldwide [1]. Cumulative evidence suggest that multifaceted process of genetic loci and environmental factors play a key role in the cancer development [2]. The well-known tumor suppressor gene p53 is involved in various cellular functions, including cell cycle arrest, apoptosis, DNA repair, and cell migration. It is mutated in various cancers [3]. The human murine double-minute gene 2 (*MDM2*, OMIM: 164785) gene is mapped to 12q14.3-15 [4]. The MDM2 protein plays an important role in cell cycle control as a  negative regulator of p53 activity. Overexpression of *MDM2* have been shown in various cancer types [5-8]. MDM2 directly binds to the p53 protein and inhibits p53 activity. In addition, MDM2 overexpression may inhibit DNA repair independent of p53 [9, 10]. Genetic variations, including single nucleotide polymorphisms (SNPs) and indel insertion/deletion (indel) polymorphisms may modify susceptibility to cancer [11-13]. A 40bp indel polymorphism (rs3730485**)** in the *MDM2* promoter P1 region, may alter the expression of *MDM2* [14]. Several studies examined the impact of *MDM2* 40bp indel polymorphism and the risk of various cancers [15-28], but the findings were inconsistent and controversial. So, we conducted an updated meta-analysis to obtain a more precise approximation of the association between this polymorphism and cancer susceptibility.

## MATERIALS AND METHODS

***Literature search:*** We performed a comprehensive search for relevant studies focusing on MDM2 40bp indel polymorphism in PubMed, Web of Science, and Scopus databases up to November 02, 2018. The search keywords were “cancer or tumor or carcinoma or neoplasms” and “MDM2 or mouse double minute 2” and “polymorphism or mutation or variant or deletion or indel or rs3730485 or del1518”. Relevant studies comprised the meta-analysis if they met the following inclusion criteria: 1) Original case-control studies; 2) studies provided sufficient genotyping data of *MDM2* 40bp indel polymorphism in both cases and controls. The exclusion criteria were: 1) case reports, conference abstract, meta-analysis, and duplication data; 2) studies lacking genotype information.

***Data extraction: ***Two investigators independently searched the databases and extracted the relevant data from eligible studies. The following data was recorded from each study including the first author, Year of publication, country, ethnicity, source of control, cancer type, genotype distributions in cases and controls and result of the Hardy-Weinberg equilibrium (HWE) test (Table 1).

**Table 1:** Characteristics of the studies eligible for meta-analysis

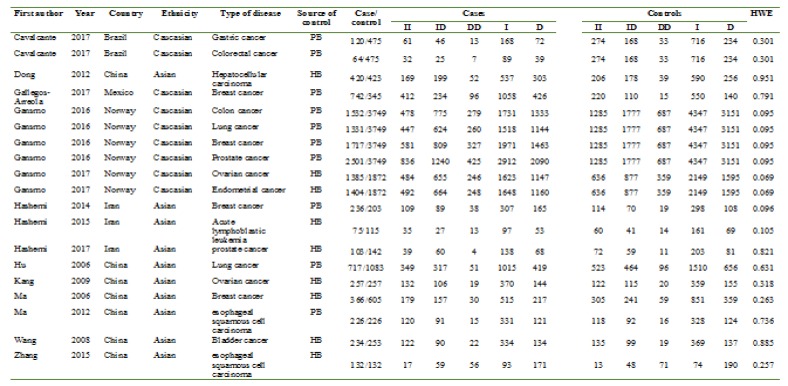

***Statistical analysis: ***All analyses were done by STATA 14.1 software (Stata Corporation, College Station, TX, USA). Departure from HWE in controls was examined by the chi-square test. The strength of the association between *MDM2* 40bp indel polymorphism and cancer risk was evaluated by pooled odds ratios (ORs) and their 95% confidence intervals (CIs). The Z-test was used for statistical significance of the pooled OR. We estimated the between-study heterogeneity by the Q-test and I^2^ test. The p<0.10 indicating the presence of heterogeneity. If heterogeneity exist, a random-effect model was employed; otherwise, a fixed-effect model was used. Stratified analyses by cancer type was also applied for each genetic comparison model. We assessed publication bias visually using funnel plots and conducting quantitative estimations with Egger’s and Begg's tests. Sensitivity analysis was executed by removing each study time to inspect the impact of individual data set on the pooled ORs.

## RESULTS

**Figure 1:**
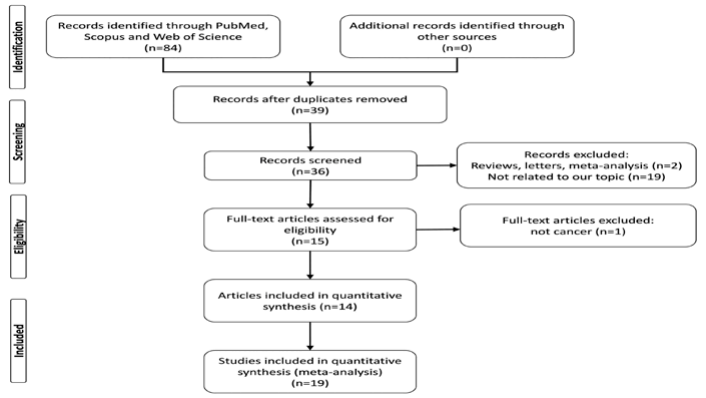
Flowchart of selection of studies for inclusion in meta-analysis

A flow chart of the study selection process is shown in Figure 1. Totally 19 case-control studies from 14 articles [15-28], including 13,562 cancer cases and 23,474 controls were included in the meta-analyses. Table 1 shows the main characteristics of the included studies.

**Figure 2:**
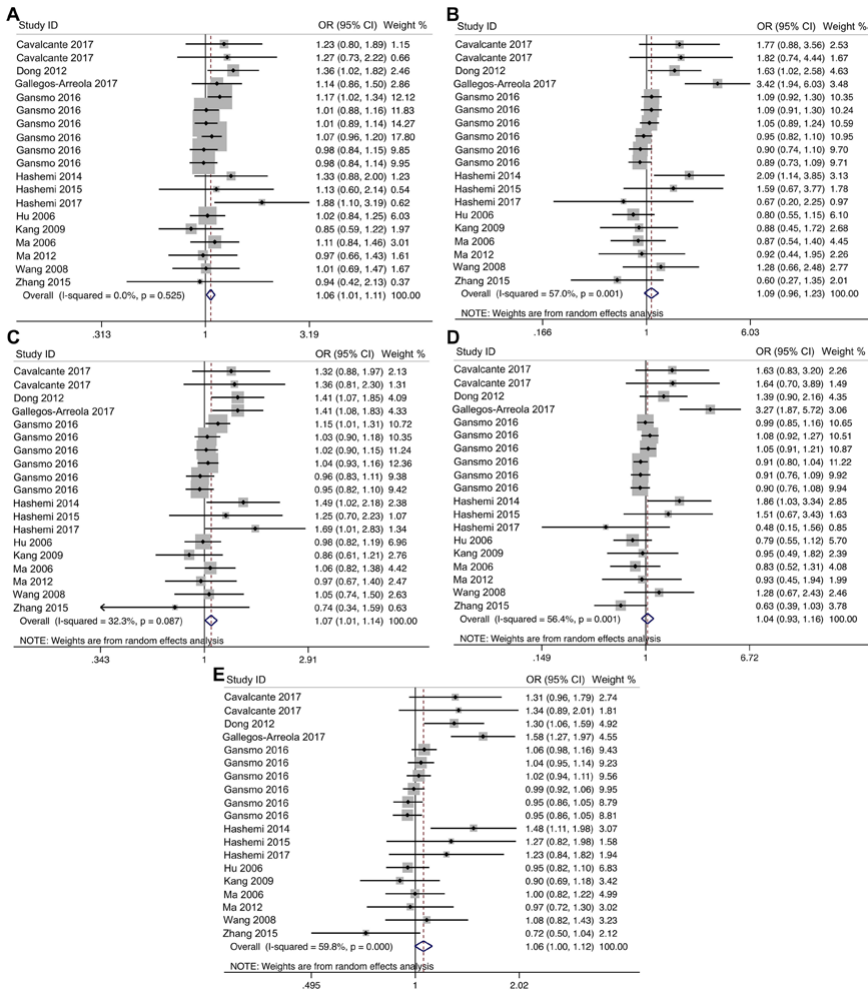
The forest plot for association between MDM2 40-bp indel polymorphism and overall cancer risk for ID *vs* II (A), DD *vs* ID (B), ID+DD *vs* II (C), DD *vs* ID+II (D) and D *vs* I (F)

The main findings of our meta-analysis and the heterogeneity test are presented in Table 2. We revealed that the polymorphism significantly associated with an increased risk of overall cancer in heterozygous (OR=1.06, 95%CI=1.01-1.11, P=0.016) and ID+DD (OR=1.07, 95%CI=1.01-1.14, P=0.027) genotypes. While no significant association between the variant and cancer risk was found in examined genetic models (Fig. 2 and Table 2). We achieved stratified analyses by cancer types (Table 2). The data showed that the polymorphism significantly increased the risk of gastrointestinal cancer in heterozygous (OR=1.18, 95%CI=1.06-1.32, P=0.003), and ID+DD (OR=1.18, 95%CI=1.06-1.30, P=0.002) genotypes (Table 2). No significant association between the indel variant and the risk of breast cancer, lung cancer, prostate cancer, ESCC, and ovarian cancer was observed. In addition, subgroup analysis by ethnicity revealed no significant association between the variant and the risk of overall cancer in Asian and Caucasian population (Table 2).

Between-study heterogeneity across studies included in the analysis is shown in Table 2. We found heterogeneity in overall comparisons between studies for homozygous codominant, recessive and allele genetic models. So random-effect model was applied for calculating ORs. Funnel plot and Egger’s test were performed to estimate the publication bias. No evidence of publication bias was detected in overall analysis (Table 2).

Sensitivity analysis was done to evaluate the stability of the findings in our meta-analysis. The sensitivity analysis revealed no obvious effects from each study in homozygous codominant, and recessive genetic models.

**Table 2:** The pooled ORs and 95%CIs for the association between *MDM2* 40-bp indel polymorphism and cancer susceptibility

**Number of stuides**		**Genetic model**s	**Association test**			**Heterogeneity test**			**Publication bias tests**	
			**OR (95%CI)**	**Z**	**P**	**χ2**	**I** ^2^ **(%)**	** P**	**Egger’s test ** ** P-value**	**Begg’s test ** **P-value**
**Overall**										
19		ID *vs* II	1.06 (1.01-1.11)	2.41	0.016	16.97	0.0	0.525	0.174	0.196
		DD *vs* II	1.09 (0.96-1.23)	1.34	0.180	41.84	57.0	0.001	0.146	0.382
		ID+DD *vs* II	1.07 (1.01-1.14)	2.22	0.027	26.60	32.3	0.087	0.105	0.382
		DD *vs* ID+II	1.04 (0.93-1.16)	0.61	0.540	41.28	56.4	0.001	0.192	0.421
		D *vs* I	1.06 (1.00-1.12)	1.84	0.066	44.76	59.8	0.008	0.092	0.132
									
**Asians**										
10		ID *vs* II	1.10 (0.99-1.23)	1.81	0.70	10.07	10.6	0.345	0.581	0.325
		DD *vs* II	1.07 (0.83-1.39)	0.55	0.586	15.14	40.6	0.087	0.905	0.929
		ID+DD *vs* II	1.10 (0.99-1.22)	1.78	0.075	13.50	33.3	0.141	0.608	0.531
		DD *vs* ID+II	1.00 (0.79-1.26)	0.01	0.991	15.31	41.2	0.083	0.680	0.929
		D *vs* I	1.06 (0.94-1.20)	0.99	0.324	18.86	52.3	0.026	0.623	0.421
									
**Caucasians**										
9		ID *vs* II	1.05 (1.00-1.11)	1.79	0.074	6.22	0.0	0.622	0.356	0.532
		DD *vs* II	1.09 (0.95-1.26)	1.20	0.231	26.62	69.9	0.001	0.029	0.211
		ID+DD *vs* II	1.05 (1.00-1.10)	1.85	0.064	12.50	36.0	0.130	0.096	0.677
		DD *vs* ID+II	1.05 (0.92-1.20)	0.76	0.448	25.93	69.1	0.001	0.028	0.095
		D *vs* I	1.05 (0.98-1.13)	1.44	0.149	25.54	68.7	0.001	0.040	0.211
									
**Gastrointestinal cancer**										
6	ID *vs* II		1.18 (1.06-1.32)	3.02	0.003	2.35	0.0	0.799	0.797	0.851
	DD *vs* II		1.14 (0.99-1.33)	1.76	0.078	7.78	35.7	0.169	0.656	0.573
	ID+DD *vs* II		1.18 (1.06-1.30)	3.09	0.002	4.95	0.0	0.422	0.902	0.348
	DD *vs* ID+II		1.02 (0.89-1.16)	0.23	0.818	8.82	43.3	0.116	0.549	0.851
	D *vs* I		1.10 (0.95-1.28)	1.28	0.202	11.22	55.4	0.047	0.867	0.851
									
**Breast cancer**										
4	ID *vs* II		1.06 (0.95-1.17)	1.05	0.293	2.14	0.0	0.544	0.016	0.042
	DD *vs* II		1.53 (0.88-2.66)	1.52	0.129	20.38	85.3	0.000	0.332	0.174
	ID+DD *vs* II		1.18 (0.98-1.42)	1.73	0.085	7.52	60.1	0.057	0.160	0.174
	DD *vs* ID+II		1.45 (0.86-2.44)	1.41	0.158	19.49	84.6	0.000	0.378	0.174
	D *vs* I		1.22 (0.97-1.53)	1.72	0.086	18.61	83.9	0.000	0.257	0.174
									
**Lung cancer**										
2	ID *vs* II		1.01 (0.90-1.14)	0.24	0.81	0.01	0.0	0.910	-	-
	DD *vs* II		0.97 (0.72-1.30)	0.20	0.84	2.26	56.0	0.130	-	-
	ID+DD *vs* II		1.02 (0.91-1.13)	0.28	0.78	0.15	0.0	0.69	-	-
	DD *vs* ID+II		0.96 (0.71-1.30)	0.26	0.80	2.59	61.0	0.11	-	-
	D *vs* I		1.01 (0.94-1.09)	0.37	0.710	1.06	6.0	0.30	-	-
									
**Prostate cancer**										
2	ID *vs* II		1.33 (0.78-2.28)	1.05	0.290	4.10	76.0	0.04	-	-
	DD *vs* II		0.95 (0.82-1.10)	0.74	0.460	0.31	0.0	0.58	-	-
	ID+DD *vs* II		1.24 (0.78-1.95)	0.91	0.360	3.26	69.0	0.07	-	-
	DD *vs* ID+II		0.87 (0.64-1.20)	0.84	0.40	1.13	11.0	0.29	-	-
	D *vs* I		1.00 (0.93-1.07)	0.07	0.95	1.20	17.0	0.27	-	-
									
**Esophageal squamous cell carcinoma**										
2	ID *vs* II		0.97 (0.68-1.37)	0.19	0.85	0.01	0.0	0.94	-	-
	DD *vs* II		0.76 (0.44-1.31)	1.00	0.32	0.57	0.0	0.45	-	-
	ID+DD *vs* II		0.92 (0.66-1.28)	0.51	0.61	0.38	0.0	0.54	-	-
	DD *vs* ID+II		0.71 (0.48-1.07)	1.64	0.10	0.75	0.0	0.39	-	-
	D *vs* I		0.86 (0.68-1.08)	1.28	0.20	1.56	36.0	0.21	-	-
									
**Ovarian cancer**										
2	ID *vs* II		0.96 (0.83-1.11)	0.56	0.57	0.50	0.0	0.48	-	-
	DD *vs* II		0.90 (0.74-1.09)	1.09	0.28	0.00	0.0	0.94	-	-
	ID+DD *vs* II		0.94 (0.82-1.08)	0.88	0.38	0.35	0.0	0.56	-	-
	DD *vs* ID+II		0.91 (0.77-1.09)	1.03	0.30	0.01	0.0	0.91	-	-
	D *vs* I		0.95 (0.86-1.04)	1.17	0.24	0.14	0.0	0.71	-	-

## DISCUSSION

The tumor suppressor p53, a transcriptional factor, really controls the growth and development of normal cells. P53, serves as an important tumor suppressor protein in preventing cancer, regulates the cell cycle and apoptosis [29-31]. Given the significant roles of MDM2 in the regulation of p53, it is biologically believable that *MDM2* polymorphism may modulate the risk of cancer. In the present study we conducted an updated meta-analysis to find out the correlation between the 40bp indel polymorphism of *MDM2* and cancer risk. Fourteen independent article [15-28] including 13,562 cancer cases and 23,474 controls investigating the genetic effects of *MDM2* 40bp indel polymorphism on cancer risk were pooled in this analysis. In our meta-analysis, 5 genetic models were considered including homozygote codominant, heterozygous codominant, dominant, recessive, and allele to evaluate the impact of *MDM2* 40bp indel polymorphism on cancer risk. The overall analysis revealed that heterozygous codominant, and dominant increased the risk of cancer. Subgroup analysis by cancer types proposed that MDM2 40bp indel polymorphism increased the risk of gastrointestinal cancer in heterozygous codominant, and dominant genetic models. No significant association was observed between the variant and the risk of breast cancer, ESCC, lung cancer, prostate cancer, and ovarian cancer, which may be due to the small number of articles.

Recently, Hua et al [32] published a meta-analysis regarding the impact of *MDM2* 40bp indel polymorphism on cancer susceptibility. They found lack of association between this polymorphism and cancer risk. One of the study they enrolled in the meta-analysis was not related to cancer [33]. In addition, the number of cases and controls in our meta-analysis is higher than that of Hua et al [32]. 

The degree of heterogeneity is an essential factor assessed in genetic association meta-analysis. In our meta-analysis, the genetic models which associated with cancer risk showed no evidence of heterogeneity. Furthermore, assessment of publication bias showed no obvious publication bias in the funnel plot under all genetic models in overall cancer as well as gastrointestinal cancer. After omitting each study in order, the pooled ORs of the remaining studies were comparable to the total pooled ORs in homozygous codominant and recessive genetic models, suggesting that the meta-analysis was stable.

Several limitations of our meta-analysis should be taken into account. First, only studies published in English were selected. Second, heterogeneity existed among the included studies. Although, the sources of heterogeneity were not clear, it may be derived from differences in cancer types and ethnicities. Third, the sample size of our meta-analysis was still relatively small in stratified analysis by cancer types (4 studies fir breast cancer; 2 studies for ESCC, lung cancer, prostate cancer, and ovarian cancer). So, the statistical power was limited. 

Despite the limitations, our meta-analysis suggest that *MDM2* 40bp indel polymorphism is a risk factor for developing overall cancer as well as gastrointestinal cancer. More well-designed large-scale case-control studies are necessary to elucidate the possible roles of this variant in cancer.
